# The efficacy and safety of isotonic and hypotonic fluids in intravenous maintenance fluid therapy in term newborns: national multicenter observational “neofluid” study

**DOI:** 10.3389/fnut.2024.1410571

**Published:** 2024-09-19

**Authors:** Hasan Ozkan, Nuray Duman, Funda Tuzun, Fatma Narter, Can Akyildiz, Emel Altuncu, Mehmet Satar, Mustafa Ozdemir, Abdullah Kurt, Ali U. Tugcu, Murat Konak, Saime Sundus Uygun, Seda Yilmaz Semerci, Rahime T. Dikmen, Bora Baysal, Cemile K. Zeybek, Yasemin Ezgi Kostekci, Suzan Sahin, Merve Tutal, Ayse Anik, Mehmet Buyuktiryaki, Belma S. Karagol, Gaffari Tunç, Derya Colak, Hasan Cetin, Aysen Orman, Ozgur Olukman, Mehmet Fatih Deveci, Dilek Sarici, Salih C. Cakir, Pembe Keskinoglu

**Affiliations:** ^1^Faculty of Medicine, Dokuz Eylül University, Izmir, Türkiye; ^2^Istanbul Kartal Dr.Lutfi Kirdar Education and Research Hospital, Istanbul, Türkiye; ^3^Faculty of Medicine, Cukurova University, Adana, Türkiye; ^4^Faculty of Medicine, Ankara Yildirim Beyazit University, Ankara, Türkiye; ^5^School of Medicine, Selcuk University, Konya, Türkiye; ^6^University of Health Sciences, Istanbul Kanuni Sultan Suleyman Training and Research Hospital, Istanbul, Türkiye; ^7^İstanbul Kanuni Sultan Süleyman Eğitim ve Araştırma Hastanesi, Istanbul, Türkiye; ^8^Faculty of Medicine, Uşak University, Usak, Türkiye; ^9^Faculty of Medicine, Ankara University, Ankara, Türkiye; ^10^Department of Pediatrics, Division of Neonatology, Aydın Maternity and Children Hospital, Aydin, Türkiye; ^11^Faculty of Medicine, Eskişehir Osmangazi University, Eskişehir, Türkiye; ^12^Medical School, Adnan Menderes University, Aydin, Türkiye; ^13^School of International Medicine, Istanbul Medipol University, Istanbul, Türkiye; ^14^Gulhane Faculty of Medicine, University of Health Sciences, Ankara, Türkiye; ^15^School of Medicine, Cumhuriyet University, Sivas, Türkiye; ^16^Umraniye Research and Training Hospital, University of Health Sciences, Istanbul, Türkiye; ^17^School of Medicine, Süleyman Demirel University, Isparta, Türkiye; ^18^Faculty of Medicine, Mersin University, Isparta, Türkiye; ^19^Faculty of Medicine, İzmir Bakircay University, İzmir, Türkiye; ^20^Faculty of Medicine, İnönü University, Malatya, Türkiye; ^21^S.B. Keçiören Eğitim ve Araştırma Hastanesi, Malatya, Türkiye; ^22^Samsun Training and Research Hospital, Samsun, Türkiye

**Keywords:** newborn, maintenance, fluid therapy, tonicity, isotonic, hypotonic, hyponatremia, hypernatremia

## Abstract

**Objective:**

The objective of this study was to evaluate the efficacy and safety of isotonic and hypotonic intravenous fluids in maintenance fluid therapy for term infants.

**Methods:**

This was a multi-centre, prospective, observational study conducted in 21 participating centres from December 30, 2020, to June 30, 2023. The study included term newborns requiring parenteral fluid therapy for maintenance (NCT04781361). The fluid treatment was divided into two groups based on the concentration of sodium in the parenteral fluid, designated as hypotonic (NaCl <130 mmol/L) and isotonic (NaCl = 130–154 mmol/L). The primary outcomes were the change in mean plasma sodium (pNa) levels per hour (∆pNa mmol/L/h), the incidence of hyponatremia (pNa <135 mmol/L) and hypernatremia (pNa >145 mmol/L), and the occurrence of clinically significant changes in sodium levels (∆pNa >0.5 mmol/L/h).

**Results:**

A total of 420 patients from 21 centers were included. The ∆pNa was negative in the hypotonic fluid group and positive in the isotonic fluid group, with a significant difference between the groups [respectively −0.07 ± 0.03 (95% CI: −0.13 to −0.02); 0.04 ± 0.03 (95%CI: −0.02 to 0.09), *p* = 0.04]. There was no difference between the groups in terms of the development of hypernatremia or a clinically meaningful pNa increase. The hypotonic fluid group had a higher incidence of hyponatremia and a clinically meaningful sodium decrease compared to the isotonic fluid group [7.9% vs. 1.2% (OR:6.5, p:0.03)] and [12.2% vs.4.2% (OR:2.9, *p* = 0.03)].

**Conclusion:**

Contrary to current understanding, this large-scale study is the first to demonstrate that the use of hypotonic fluids in maintenance fluid therapy for newborns poses a risk of hyponatremia development, whereas isotonic fluid therapy appears safe.

## Introduction

Maintenance fluid therapy is defined as the volume of fluid required to meet daily metabolic needs, such as normal water and electrolyte losses, and maintain homeostasis (1). In children, maintenance intravenous fluid therapy aims to replace fluid and electrolyte losses under normal homeostatic conditions. The intermediate byproducts formed as a result of metabolism are heat and solutes, and these must be removed from the body for homeostasis. Heat is excreted through the skin and respiration, while solutes are excreted through urine. Therefore, maintenance fluid therapy should account for both sensible losses (urine and feces) and insensible losses (skin and lungs). For many years, fluid and electrolyte requirements in maintenance fluid therapy were calculated as suggested by Holliday and Segar. Traditionally, hypotonic fluids have been used for maintenance fluid therapy for infants, considering the daily energy needs of healthy infants and the sodium content of breast milk (2).

Recent studies have shown an increased risk of hyponatremia when hypotonic fluids are used for maintenance fluid therapy in children compared with isotonic fluids (3–6). As a result, relevant pediatric guidelines now recommend the use of isotonic fluids rather than hypotonic fluids for maintenance intravenous fluid therapy in children (7–10). In neonates, hypotonic fluids are still used for maintenance intravenous fluid therapy, and there is no consensus on the ideal fluid composition. Since there is no prospective, observational, multicenter study investigating the effectiveness and reliability of isotonic and hypotonic fluids in intravenous maintenance fluid treatment in term newborn babies, term newborn babies were included in this study.

Fluid and electrolyte needs in preterm babies vary greatly according to gestational age and postnatal age, and in addition to physiological maturity, nutritional needs and environmental factors also play an important role in maintenance fluid therapy. Since individualized maintenance fluid therapy would be a more appropriate approach for these babies, these babies were excluded from the study.

In 2015, the National Institute for Health and Clinical Excellence (NICE) published the “Intravenous Fluid and Electrolyte Treatment Guideline for Newborns and Children,” which recommended the use of isotonic fluids for the maintenance fluid therapy in newborns after the postnatal renal adaptation period (8). The 2020 version of this guideline emphasized the postnatal day and recommended isotonic maintenance fluids (5–10% glucose and 131–154 mmol/liter NaCl) for term neonates after the 8^th^ postnatal day. For term neonates up to 7 days of age, it is suggested that professional judgment be used, taking into account individual circumstances highlighting the possibility of higher sodium content and lower glucose content in these fluids. It is important to note that NICE recommendations on fluid therapy in newborns were based on expert opinion rather than evidence, as there were few trials available on this topic (9). Since this guideline is not based on strong evidence, it has not yet made a significant change in clinical practice regarding the maintenance fluid therapy of newborn babies. The objective of this study was to assess the efficacy and safety of isotonic and hypotonic fluids as intravenous maintenance fluids in neonates through a prospective, observational, multicenter study.

## Method

This prospective multicenter observational study was conducted from December 30, 2020, to June 30, 2023, with protocol registered on ClinicalTrials.gov (NCT04781361, February 2021). The primary outcome was the hourly change in plasma sodium level (∆pNa, mmol/L/h) during the first day of isotonic and hypotonic fluid therapy. Secondary outcomes included hyponatremia (plasma sodium <135 mmol/L), hypernatremia (>145 mmol/L), critical ∆pNa (>0.5 mmol/L/h), and treatment-related morbidities such as intraventricular hemorrhage, edema, apnea, seizures, hypertension, tachycardia, bradycardia, electrolyte imbalances, renal dysfunction, length of hospital stay, and mortality.

The inclusion criteria were as follows: (i) born at 37 weeks’ gestation or later; (ii) completed the first 24 h of life and were less than 30 days old; (iii) had a Na level between 135 and 145 mmol/L; (iv) were receiving at least 50% of daily fluid requirements intravenously as prescribed by the attending physician; and (v) had written consent from the family or legal guardian. Neonates with severe dehydration, shock, cardiac or hepatic failure, metabolic disease, renal failure, adrenal insufficiency, diabetes mellitus, diabetes insipidus, hypoxic ischemic encephalopathy, congenital anomalies, diuretic therapy, significant edema, those receiving total parenteral nutrition therapy, and patients receiving non-standard fluid therapy were excluded from the study.

Each participating center submitted its case data to the Trials Network online system. All entries on the Trials Network (https://www.trials-network.org/neonatology) were rigorously screened for adherence to the inclusion/exclusion criteria. The study used an online case report form as the primary tool for documenting demographic data (postnatal age, mode of delivery, gestational age, birth weight, sex, diet) and clinical information. Case report form was designed for optimal collection of data in accordance with the study protocol and standardized to the needs of all those who handle the data such as investigator, biostatistician, clinical research monitor/coordinator, and data entry personnel according to the standart guidelines (11).

Clinical data, recorded daily for up to 72 h during intravenous fluid therapy, included primary diagnosis’ ICD codes, baseline body weight, percentage weight loss, clinical hydration status, blood pressure, heart rate, parenteral fluid type, sodium content (mmol/L), daily intravenous fluid intake, daily enteral fluid volume and composition, medications for the primary illness, and daily urine and stool counts. Morbidities potentially influenced by fluid therapy, such as intraventricular hemorrhage, edema, apnea, and seizures, as well as morbidities related to the primary disease, mortality, and length of stay, were recorded.

Throughout the study, serum blood sugar, blood urea nitrogen, creatinine, electrolytes (sodium, potassium, chloride), pH, bicarbonate, urine density, and spot urine sodium and creatinine levels were monitored in the cases before treatment, and at 24, 48, and 72 h of treatment if fluid therapy was ongoing. Monitoring was also conducted when fluid therapy was discontinued or in the presence of severe hyponatremia (< 135 mmol/L) or severe hypernatremia (> 145 mmol/L), with at least a 3 mmol/L change from baseline during therapy, or when the clinician decided to change the treatment due to the baby’s worsening clinical condition.

### Sample size

Given the absence of any studies with a similar design in the literature, a power analysis using Cohen’s t-test was conducted to determine the appropriate sample size. With an effect size of 0.5, a power of 95%, and a confidence interval of 95%, it was determined that each group would require 80 participants. Either group provides the predetermined sample size. Additional cases were included for adequate subgroup representation.

### Statistical analysis

Administered fluids were categorized as hypotonic (<130 mmol/L NaCl) or isotonic (130–154 mmol/L NaCl), excluding sodium-free fluids. Data from both groups were subjected to the Kolmogorov–Smirnov test for normality. The t-test was used for normally distributed variables, the Mann–Whitney U test for non-normally distributed variables, and chi-squared analysis for categorical data. Subgroup analysis was performed using the Kruskal-Wallis test. Statistical significance was set at 0.05. Analyses were performed using IBM SPSS Statistics 25 software. If laboratory data for primary outcomes were missing, those infants were excluded. However, the sample size required for the primary outcome was still met. A linear mixed-effects model was employed using the PROC MIXED procedure in SAS to investigate the effects of time and treatment group on the outcome variable, “Na” (SAS software, Version 9.3; SAS Institute, Cary, NC, ABD). The model included fixed effects for group (hipotonic and isotonic), time, and their interaction allowing us to assess both the main effects and the interaction between these factors.

### Ethical approval statement

The study was approved by both the Turkish Medicines and Medical Devices Agency of the Ministry of Health (TITCK, 17-AKD-174) and the Interventional Clinical Research Ethics Committee of Dokuz Eylul University (404-SBKAEK). It was conducted in strict accordance with the ethical standards of the 1964 Declaration of Helsinki and its subsequent amendments. The Turkish Society of Neonatology Online Studies Scientific Steering Committee approved the study protocol.

### Data privacy

Each center reported the data of its own cases to the Trial Network web-based data recording system “Trials Network” (https://www.trials-network.org/neonatology) using a unique identification number. When the study coordinator needed to verify the data of any case, the responsible investigator at the center accessed the patient identification data through this unique number and verified it. During the study period, cases entered into the Trials Network were checked for inclusion and exclusion criteria, and monthly correction emails were sent to the centers. Corrected cases were transferred to the SPSS system after each check. Centers that logged into the system could only view their own data. Only the research coordinator had access to the aggregated, non-identifying data.

## Results

**Figure 1:**
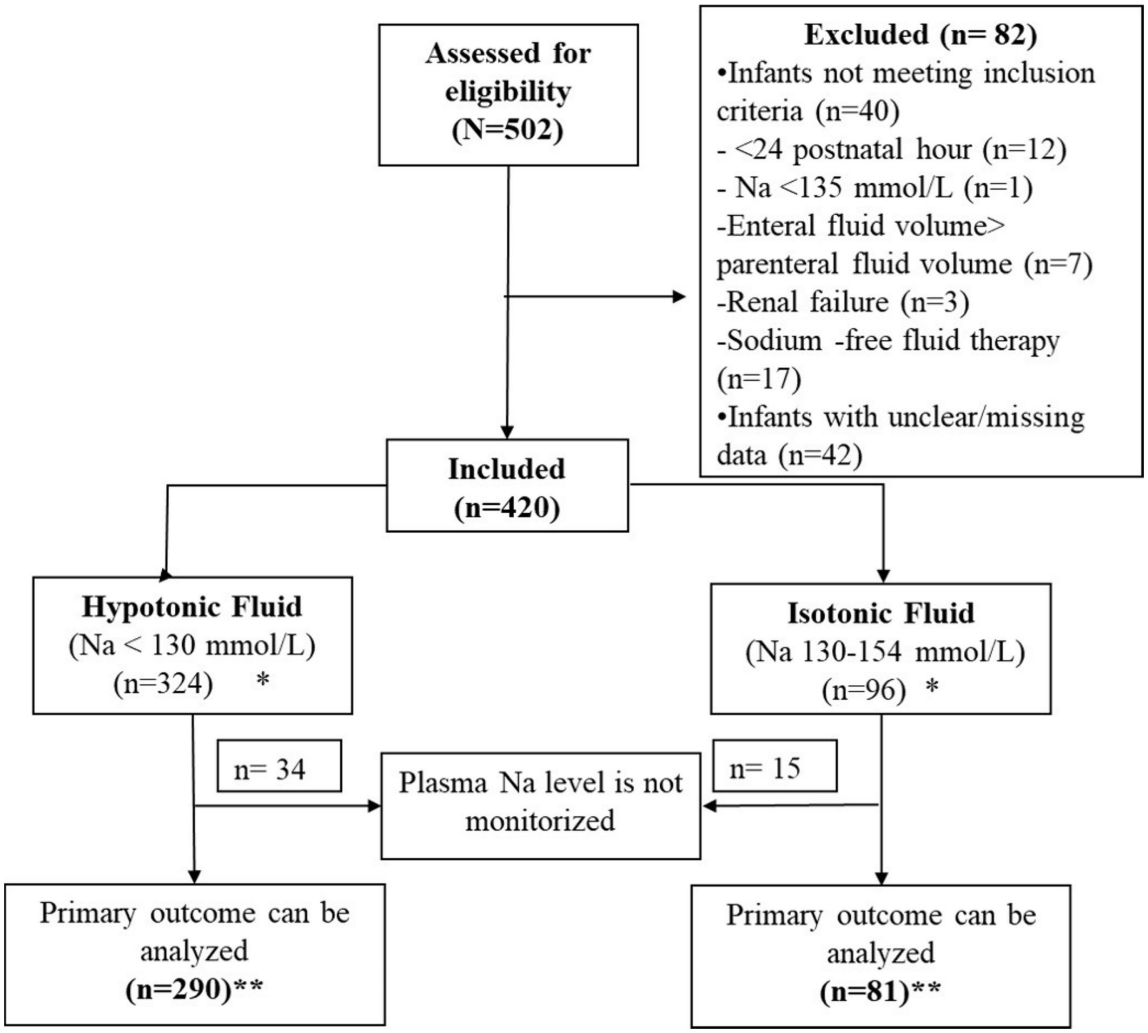
Flow diagram. *Demographic data and treatment-related morbidities were evaluated in 420 patients. **As the primary evaluated outcome was the hourly change in plasma sodium level (∆pNa), cases without follow-up sodium levels were excluded from the primary outcome analysis.

Data was collected between December 30, 2020, and June 30, 2023, from 21 participating centers, resulting in a total of 502 patients being reported. After excluding 82 infants, 420 infants were included. The excluded infants were as follows: 42 patients were excluded due to incorrect or missing data, 12 patients were excluded because they initiated fluid therapy within the first 24 h of the postnatal period, 1 patient was excluded due to a pre-treatment sodium value of less than 135 mmol/L, 7 patients were excluded because the planned daily enteral volume was greater than the parenteral volume, 3 patients were excluded due to renal failure, and 17 patients were excluded because they received a sodium-free solution containing only dextrose (Figure 1). Cases excluded from the study did not cause bias because they did not affect the predetermined sample size in both groups.

According to the protocols of the participating units, a total of 324 patients received hypotonic fluid therapy, while 96 patients received isotonic fluid therapy. There were no significant differences between the groups for demographic and clinical variables (*p* > 0.05), except feeding status at the initiation of the fluid therapy. Almost half of the cases included in the study were diagnosed with feeding problems, transient tachypnea of the newborn and neonatal jaundice. Pretreatment laboratory values were also similar (*p* > 0.05) (Table 1).

**Table 1:** Demographic and pre-treatment data of the patients by maintenance parenteral fluid type*.

	Hypotonic fluid (<130 mmol/L) (*n*:324)	Isotonic fluid (130-154 mmol/L) (*n*:96)	*p*
**Demographic data**			
Gestational age (days)	271.4 ± 8.3	270.6 ± 0.7	0.446
Birth weight (gr)	3268 ± 29	3225 ± 45	0.449
Cesarean delivery	187 (57.7%)	58 (60.4%)	0.724
Male gender	202 (62.3%)	55 (57.3%)	0.405
**Nutrition at the time of admission**			
Not fed Exclusive breast milk Formula Breast milk + formula	34 (10.5%) 145 (44.8%) 47 (14.5%) 98 (30.2%)	1 (1.0%) 66 (68.8%) 5 (5.2%) 24 (25.0%)	<0.001**
**Fluid therapy data**			
Postnatal age at which fluid therapy was started (hours)	96 ± 8	121 ± 16	0.130
Body weight (grams) at the start of fluid therapy	3207 ± 30	3139 ± 46	0.265
Planned total daily fluid volume (cc/kg/day)	106 ± 2	114 ± 4	0.139
Planned total daily parenteral fluid volume (cc/kg/day)	76 ± 2	83 ± 3	0.139
Planned total daily enteral fluid volume (cc/kg/day)	31 ± 1	30 ± 2	0.816
**Pre-treatment laboratory values**			
BUN (mg/dl)	13.6 ± 0.7	13.1 ± 1.0	0.748
Creatinine (mg/dl)	0.69 ± 0.01	0.60 ± 0.03	0.07
Sodium (mmol/L)	139.9 ± 0.2	139.8 ± 0.3	0.654
Potassium (mmol/ L)	4.8 ± 0.4	4.9 ± 0.1	0.401
Chlorine (mmol/L)	106.0 ± 0.3	107.0 ± 0.7	0.134
Glucose (mg/dl)	78.4 ± 1.1	77.0 ± 2.0	0.552
Serum pH	7.37 ± 0.01	7.35 ± 0.02	0.096
Serum HCO _3_ (mmol/L)	22.2 ± 0.2	21.7 ± 0.8	0.455
Urine Sodium (mmol/L)	32.2 ± 4.3	41.1 ± 9.0	0.414
Urine Creatinine (mg/dl)	30.7 ± 6.0	18.1 ± 3.6	0.379
Urine pH	6.14 ± 0.06	6.20 ± 0.14	0.691
Urine Density	1009 ± 1	1009 ± 1	0.963
**Urinary findings after fluid therapy**			
Urine Sodium (mmol/L)	26.6 ± 3.5	38.2 ± 8.6	0.210

*Data are given as mean ± SD or number (%) as applicable.

**The differences were significant in all subgroups.

**Figure 2:**
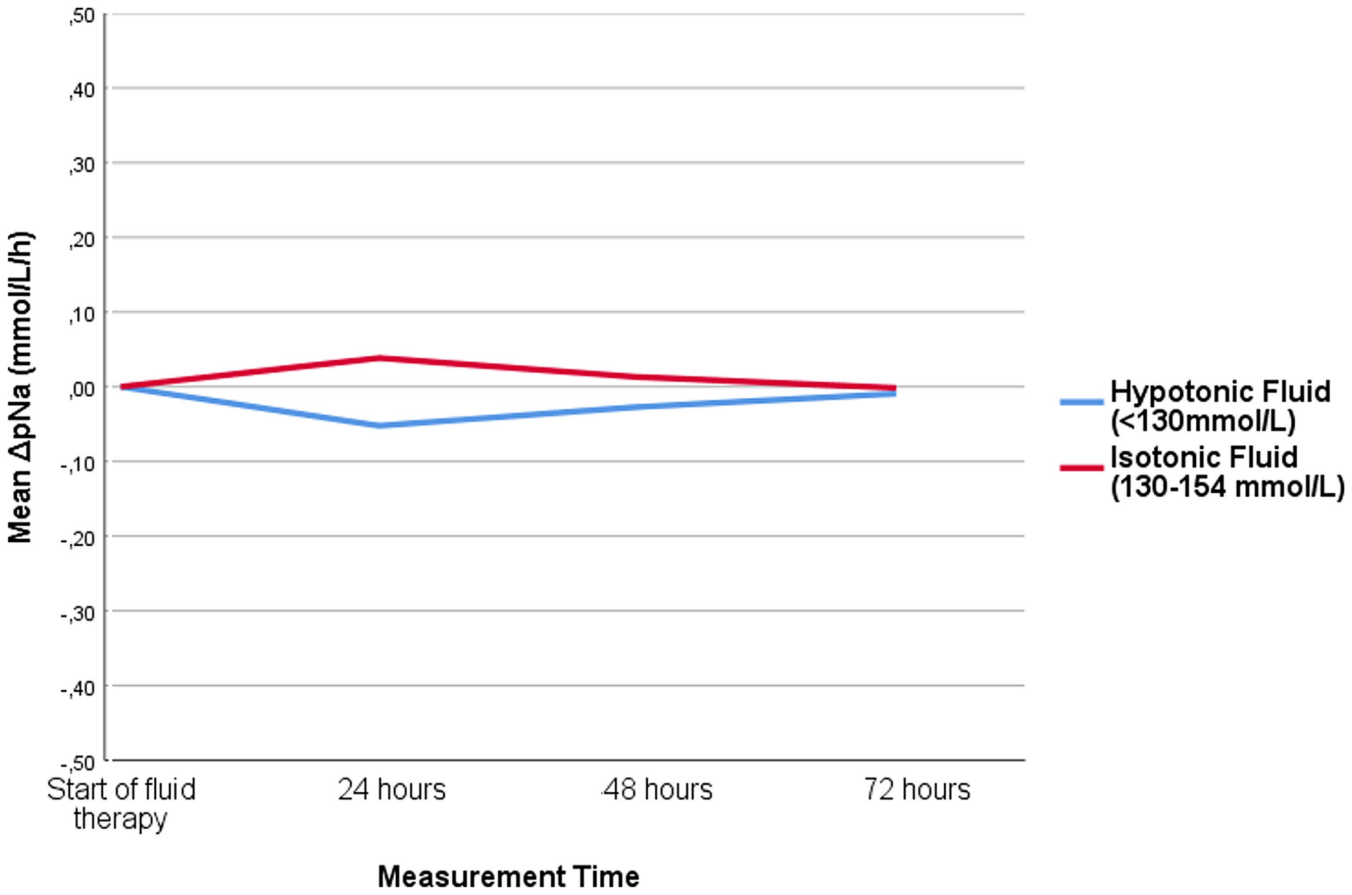
Hourly change in plasma sodium level of patients receiving isotonic and hypotonic fluid.

Measurement of sodium levels within the first 24 h of fluid therapy was conducted in a total of 371 patients, with 290 receiving hypotonic fluid and 81 receiving isotonic fluid. When comparing ∆pNa (mmol/L/h) between the groups as the primary outcome, the hypotonic fluid group showed a negative change [−0.07 ± 0.03, 95% CI: −0.13 to −0.02]. In contrast the isotonic group demonstrated a positive change [0.04 ± 0.03, 95% CI: −0.02 to 0.09] (*p* = 0.04). Considering the distinct initial feeding types in the isotonic and hypotonic fluid groups, we evaluated the impact of feeding type on ∆pNa. Results showed no significant difference in median ∆pNa between subgroups categorized by infants’ initial feeding type (Kruskal-Wallis test, *p* = 0.99). When a linear mixed-effects model study was used to evaluate differences over time between two treatments, the interaction between time and group was not statistically significant [*F* (1,118) = 2.06, *p* = 0.1534], implying that the trajectory of the outcome variable over time did not differ significantly between the groups (Figure 2).

For patients who continued fluid therapy beyond 24 h, a separate evaluation was performed between 24 and 48 h, involving 141 patients. Among these, 89 received hypotonic fluid and 52 received isotonic fluid. No significant differences were observed in terms of ∆pNa. Similarly, 15 patients in each group received fluid treatment between 48 and 72 h and there was no difference between the groups in terms of evaluated outcomes.

Furthermore, when the fluid treatments were categorized as extremely hypotonic (containing 20–50 mmol/L Na), moderately hypotonic (containing 60–130 mmol/L Na) and isotonic (containing 154 mmol/L Na), no significant difference in ∆pNa was observed between the groups.

To assess secondary outcomes, we analyzed cases with hyponatremia and hypernatremia at 0–24 h, 24–48 h and 48–72 h of fluid therapy. We also evaluated cases with ∆pNa greater than 0.5 mmol/L (increase or decrease). The results showed a higher incidence of hyponatraemia in the first 24 h in hypotonic fluid recipients compared with isotonic fluid recipients. In addition, the hypotonic fluid group had a sodium decrease of >0.5 mmol/L/h. No significant between-group difference was found for hypernatremia or sodium increase (Table 2).

**Table 2:** Secondary outcomes related with plasma sodium level*.

	Hypotonic fluid (<130 mmol/L) *n* = 290	Isotonic fluid (130-154 mmol/L) *n* = 81	OR	*p*
**Within 24 h of fluid therapy**				
Hyponatremia <135 mmol**/L**	23 (7.9%)	1 (1.2%)	6.5	0.03
Hypernatremia >145 mmol**/L**	12 (4.1%)	2 (2.5%)	–	0.74
Na increase rate ≥ 0.5 mmol**/L/h**	14 (8.9%)	6 (11.5%)	–	0.57
Na decrease rate ≥ 0.5 **mmol/L/h**	23 (12.2%)	1 (4.2%)	2.9	0.03

	Hypotonic fluid (<130 mmol/L) *n* = 89	Isotonic fluid (130-154 mmol/L) *n* = 52	OR	*p*
**Within 24–48 h of fluid therapy**				
Hyponatremia <135 mmol**/L**	7 (7.9%)	1 (1.9%)	–	0.25
Hypernatremia >145 mmol**/L**	3 (2.1%)	0 (0.0%)	–	0.29
Na increase rate ≥ 0.5 **mmol/L/h**	0 (0.0%)	0 (0.0%)	–	–
Na decrease rate ≥ 0.5 **mmol/L/h**	0 (0.0%)	0 (0.0%)	–	–

*Data are given as number (%).

–Odds ratio is not applicable for the corresponding outcome.

To analyze the effect of postnatal age, the study compared the risk of hyponatremia and hypernatremia between those treated within 24–72 h of birth and those treated after 72 h. Cases within 72 h had a higher incidence of hyponatremia [22 cases (8.6%) vs. 2 cases (1.7%), RR: 0.73, 95% CI: 0.64–0.85, p: 0.01]. In addition, the association between parenteral fluid volume in the first 24 h of therapy and the development of hyponatraemia was assessed, but no significant difference was found. Serum pH changes were compared between hypotonic and isotonic fluid recipients during the first 24 h of therapy and at baseline, with no significant difference between the groups.

As no other parameter was found to significantly influence the change in plasma sodium levels, logistic regression analyses were not performed.

## Discussion

This study suggests that isotonic fluids may be a more appropriate choice for intravenous fluid maintenance therapy in term neonates who have completed the postnatal adaptation phase. Hypotonic fluids, which are traditionally used in maintenance therapy, were found to decrease serum sodium levels and cause hyponatremia. In contrast, isotonic fluids did not cause a significant increase in serum sodium levels or hypernatremia.

As seen in Table 1, there were nutritional differences between the two groups at the beginning of the study, but this did not cause any difference in laboratory data of the two groups before treatment. During the treatment, enteral and parenteral fluid intakes of both groups were planned to be similar. Therefore, the only factor affecting the primary and secondary results of the study was the use of hypotonic or isotonic fluid.

Adaptation to extrauterine life consists of three phases of fluid balance. After birth, there is an efflux of fluid from the intracellular to the extracellular compartment. This results in salt and water diuresis by 48–72 h of age. Loss of this excess extracellular fluid (ECF) and evaporative water loss from immature skin results in physiological weight loss during the first week of life. The first transitional period ends with maximum weight loss. The second intermediate phase is characterized by a decrease in insensible water loss with increasing maturation of the skin barrier, a decrease in urine volume to less than 1–2 mL/kg per hour and low sodium excretion. The third phase consists of stable growth and is characterised by continuous weight gain with a positive net balance for water and sodium. This issue is addressed in the NICE guideline as the completion of the postnatal adaptation phase of the initiation of postnatal diuresis and weight loss in term newborn babies (9). In this study, the postnatal adaptation phase is accepted the first phase of the first 48–72 h of life when diuresis begins. Therefore, both fluid groups were considered to have completed this phase.

Limited studies have questioned the maintenance of fluid management in neonates. Balasubramanian et al. found that the use of hypotonic fluids increased the risk of mild hyponatremia, whereas the use of isotonic fluids increased the risk of hypernatremia, and they suggested the use of a fluid solution with a salt content between 0.9 and 0.2% (12). However, Moritz et al. stated that isotonic fluids did not cause hypernatremia and that such a result may be due to the inclusion of hypernatremic patients and the fact that isotonic fluids did not make a significant difference in sodium levels (13). They emphasized that when isotonic fluid is administered in the presence of hypovolemia, the fluid will be retained until the volume depletion is corrected and will not result in a significant fall in serum sodium. Once the volume depletion is corrected, further fluid administration will result in natriuresis and free water will be generated to prevent the development of hypernatremia (14).

Tüzün et al. conducted a retrospective cohort study involving 108 neonates comparing 5% dextrose in 0.9% sodium chloride (NaCl) with 5% dextrose in 0.45% NaCl for maintenance fluid therapy (15, 16). They found that hypotonic fluids could critically lower serum sodium levels, whereas isotonic fluids did not lead to hypernatremia or other complications. The authors attributed this non-existent hypernatremia to the ability of neonates to excrete sodium in the urine after physiological adaptation.

Dathan et al. conducted a study comparing isotonic (sodium chloride 0.9% and dextrose 5%) and hypotonic (sodium chloride 0.15% and dextrose 5%) fluids for intravenous maintenance therapy in neonates over 34 weeks’ gestation (17). They observed a higher incidence of hypernatremia in the isotonic fluid group, without showing superiority in preventing hyponatremia over hypotonic fluids. The authors attributed this to factors such as sodium load, limited urinary sodium excretion due to lower glomerular filtration rate and higher tubular sodium reabsorption, and the infants’ medical conditions. In particular, the study included preterm infants older than 34 weeks with a variety of medical conditions that affect fluid-electrolyte balance. It’s important to take these differences into account, including differences in fluid volume, when comparing results with our study.

In term neonates, the glomerular filtration rate is low and the ability of the newborn kidney to concentrate urine is limited but can dilute it (50–600 mOsm/kg) due to the immaturity of the luminal membrane Na-H exchanger, Na-glucose cotransporter and Na-K-ATPase (18–21). However, even healthy preterm infants can tolerate fluid intake of up to 200 mL/kg/day after the physiological adaptation period by excreting it in the urine (22). Furthermore, extracellular volume expansion has a natriuretic effect independently of glomerular filtration rate and it should be noted that various hormonal influences play a role in maintaining water and electrolyte homeostasis despite significant variations in intake (23). Factors such as desalination and translocational hyponatraemia should be taken into account in maintenance fluid therapy (24). The process of desalination involves the administration of isotonic solutions and the development of hyponatremia due to the loss of hypertonic urine. The antidiuretic hormone, with the associated suppression of aldosterone, causes the retention of electrolyte-free water formed as a result of hypertonic urine loss. Hyponatraemia due to desalination is rarely seen when isotonic fluids are administered postoperatively. In conditions such as hyperglycaemia or hypertriglycaemia, the osmotic gradient causes free fluid to move from the intracellular to the extracellular compartment. This leads to a decrease in the serum sodium concentration in the extracellular fluid. This is known as translocational hyponatraemia. However, desalination or translocational hyponatremia wasn’t seen in cases included this study.

The metabolic rate of newborn babies is 20–30% lower than that of older infants during the first 10 days. Therefore, when determining fluid and electrolyte requirements based on weight, higher amounts are calculated. Based on the literature and a thorough evaluation and interpretation of current parenteral and enteral feeding guidelines, there are insufficient data to determine the optimal composition and timing of nutritional support in preterm and term critically ill neonates (25). Studies of resting energy expenditure (REE) during the first weeks of life in healthy preterm and term infants show that REE increases with increasing energy supply during the first weeks of life, and that REE is directly proportional to growth rate. In addition, the Holliday-Segar nomogram allocates approximately 50% of energy expenditure to growth, which may not be applicable to hospitalized infants. Therefore, during acute illness or in the postoperative period, the combined fluid loss (both sensible and insensible) may be approximately half of that suggested by Holliday and Segar (50–60 mL/kg/day) (26). Factors such as pain, stress, fever, infection, hypoxia and surgery can contribute to non-osmotic inappropriate secretion of antidiuretic hormone (ADH) (21). Traditional neonatal fluid therapy often neglects these aspects and overlooks the central role of tonicity in water-electrolyte balance, which is determined by plasma sodium levels. Tonicity, which is significantly influenced by intracellular potassium, controls fluid movement between compartments (26).

There are limitations to consider. The inability to randomize participants is a major drawback. The inclusion of only infants without serious health problems requiring maintenance therapy limits the generalisability of the results to the whole neonatal population. The results of this study cannot be generalized to the excluded infants, which include preterm infants, infants with severe dehydration, shock, heart or liver failure, metabolic diseases, renal failure, adrenal insufficiency, diabetes mellitus, diabetes insipidus, hypoxic–ischemic encephalopathy, congenital anomalies, significant edema, and those in the postoperative period. Excluding these 82 infants was necessary to maintain the integrity and consistency of the study data. However, it is important to note that these exclusions did not affect the predetermined sample size for both groups, thereby helping to mitigate concerns about significant bias. Although the subjects were monitored for 72 h, the number of infants requiring fluid therapy and the measured data showed a marked decrease over time. Plasma ADH levels, plasma and urine osmolality and tonicity were not measured in this study. In addition, the postnatal physiological adaptation period was clinically defined solely by the onset of urine output in the infants.

In conclusion, this study has provided evidence for the efficacy and safety of isotonic fluids for maintenance therapy in term neonates who have completed the postnatal adaptation period. In these babies, we can initially use isotonic crystalloids containing sodium in the range of 131–154 mmol/litre with 5–10% glucose. However, the neonate’s age, diagnosis, fluid-electrolyte balance and medications should be taken into account, and serum electrolytes should be closely monitored during intravenous fluid therapy. When selecting intravenous fluids, it is important to consider not only sodium but also the individual electrolyte and glucose requirements of the newborn. If the results of this study are supported by other studies, hypotonic fluids, which have traditionally been used for maintenance fluid therapy in term infants, will be replaced by isotonic fluids.

### What is known

- Traditional practice employs hypotonic fluids for maintenance intravenous therapy in newborns, lacking a definitive fluid composition consensus beyond NICE guidelines.

### What is new

- Traditional use of hypotonic fluids in maintenance therapy led to reduced serum sodium levels, resulting in hyponatremia.
- Isotonic fluids could be a better option for intravenous maintenance fluid therapy in term newborns after the postnatal adaptation phase without causing significant serum sodium changes or hypernatremia.

## Data Availability

The datasets presented in this study can be found in online repositories. The names of the repository/repositories and accession number(s) can be found in the article/Supplementary material.
